# Validated reference genes for normalization of RT-qPCR in developing organs of wheat to study developmentally/spatio-temporally expressed family genes

**DOI:** 10.1038/s41598-025-08295-6

**Published:** 2025-07-02

**Authors:** Joanna Bocian, Bartosz Jabłoński, Anna Nadolska-Orczyk

**Affiliations:** Department of Functional Genomics, Plant Breeding and Acclimatization Institute-National Research Institute, Radzikow, Blonie, 05-870 Poland

**Keywords:** Biotechnology, Genetics, Molecular biology, Plant sciences

## Abstract

Quantitative real-time PCR (RT-qPCR) is one of the most accurate methods for gene expression analysis. An important step in such an examination is normalization of the results by the application of appropriate internal control. In this study, we evaluated ten candidate reference genes in wheat (*Triticum aestivum*) to identify the most stable for normalization across different tissues and organs of developing plants. In Experiment 1, ten reference genes were analyzed in three tissues of the Ostka cultivar, with stability rankings generated using BestKeeper, NormFinder, geNorm, and RefFinder. Among these, *Ta2776*, *eF1a*, *Cyclophilin*, *Ta3006*, *Ta14126*, and *Ref 2* were consistently identified as the most stable, while *β-tubulin*, *CPD*, and *GAPDH* were the least stable. In Experiment 2, six reference genes were tested across five tissues. *Ta2776*, *Cyclophilin*, *Ta3006*, and *Ref 2* showed high stability across tissues, whereas *CPD* and *Actin* were less reliable. Analysis of the two best performing genes, *Ref 2* and *Ta3006*, in twelve tissues/organs from two wheat cultivars, Kontesa and Ostka, revealed no significant differences in their expression between cultivars, confirming their suitability as reference genes for broader studies. Expression analysis of two target, developmentally expressed genes, *TaIPT1* and *TaIPT5*, was conducted using absolute and normalized values. For *TaIPT1*, expressed in developing spikes, normalized and absolute values showed no significant differences. In contrast, for *TaIPT5*, expressed across all tested tissues, significant differences were observed between absolute and normalized values in most tissues. However, normalization using *Ref 2*, *Ta3006*, or both reference genes produced consistent results, underscoring the importance of proper reference gene selection. This study highlights the reliability of *Ref 2* and *Ta3006* as reference genes for accurate normalization in wheat gene expression analyses, providing a robust framework for future research.

**Supplementary Information:**

The online version contains supplementary material available at 10.1038/s41598-025-08295-6.

## Introduction

Common wheat (*Triticum aestivum* L.; AABBDD) is a vital cereal crop, widely cultivated around the world. Its grains are rich in essential nutrients such as carbohydrates, proteins, vitamins, and minerals, which are crucial for feeding the growing population^1,2^. Ensuring continuous improvement in the productivity and seed quality of this crop is crucial, especially in the context of a changing environment^3,4^.

Advances in reference genome databases for wheat^5,6^combined with molecular and genetic engineering techniques, provide breeders with powerful tools to define gene functions, identify genes with the potential to enhance agricultural traits, and select them for breeding programs. Targeted genome modification techniques enable the precise incorporation of such genes into breeding lines, facilitating the development of agronomically superior varieties^7,8^.

Developmentally regulated genes, such as cytokinin metabolite genes, often belong to gene families, including transcription factors that regulate their expression^9–12^. Many of these genes are specifically expressed in developing organs or tissues, while others are expressed spatiotemporally across different tissues throughout the plant’s life cycle^13^. Additionally, in allohexaploid wheat, most genes have homoeologs in the A, B, and D genomes, which may perform distinct functions and contribute to agronomic traits^14–17^.

The initial step in characterizing the functions of gene families is to investigate their expression patterns in developing plants^18^. Quantitative real-time PCR (RT-qPCR) remains one of the most sensitive and reliable techniques for gene expression analysis in plants. However, its accuracy critically depends on the use of stable internal reference genes to normalize target gene expression across different tissues, developmental stages, or experimental conditions. Inappropriate or unstable reference genes can lead to misleading results, particularly in complex polyploid species such as wheat, where gene copy number variation and homoeologous expression further complicate normalization^19–22^. To address this, several statistical algorithms have been developed to evaluate the expression stability of candidate reference genes. Among the most widely used are **geNorm**^20^which calculates gene stability based on the average pairwise variation between genes; **NormFinder**^23^which considers both intra- and inter-group variation; **BestKeeper**^24^which uses standard deviation and coefficient of variation of Ct values; and **RefFinder**^25^an online tool that integrates the results of multiple algorithms to produce a comprehensive ranking. Employing multiple methods allows for a more reliable selection of reference genes, as each algorithm emphasizes different aspects of expression stability.

Paolacci et al.^21^ conducted the first extensive screening of reference genes for RT-qPCR normalization in different wheat tissues. Their study tested the stability of 32 genes across 24 samples and identified three new reference genes that were more stable than traditional housekeeping genes, such as genes encoding actin or α-tubulin proteins. Subsequent genome-wide studies of reference genes in wheat evaluated different tissues, developmental stages, and environmental conditions^26^and extended this analysis to other Triticeae species to explore orthologous genes through comparative transcriptomics^27^. However, most studies on reference gene identification have focused on specific developmental processes or stress responses. These include studies on endosperm development^28^grain filling^29^flag leaves under varying farming conditions^30^and wheat microsporogenesis and meiosis^31,32^. Other research has addressed biotic and abiotic stress, such as drought and salt stress^33^short-term drought stress^34^studying viral infections in cereals^35^rust infections in wheat^36^studying *Puccinia coronata* or *Puccinia graminis* interaction with *Avena sativa*^37,38^and the quantification of microRNA expression following stress treatments^39^. The results of selection of the most stable reference genes were dependent on the experimental system, environmental conditions, species specificity, and their interaction with pathogens.

In our research on the specificity of expression of wheat *cytokinin oxidase/dehydrogenase* (*TaCKX*) family genes in different organs of developing wheat plants^13^we pre-selected (not reported) reference genes based on the work of Paolacci et al.^21^identifying *ADP-ribosylation factor* (*Ref 2*) as the most suitable one. This gene was also used in subsequent experiments to study the functions of the *TaCKX1* and *TaCKX2* genes^40–42^and *NAC* genes^43^. In addition to us, some researchers have already proven that one reference gene is optimal for use in the study of wheat grain filling^29^. However, some researchers have opted to use two reference genes to obtain more accurate results. In line with these findings, and before investigating the specificity of expression of wheat *isopentenyltransferase* (*TaIPT*) genes in different organs of developing wheat plants, we analyzed additional potential candidates as reference genes. Selected in our new experimental system the most stable reference genes were applied to compare the expression of two *TaIPT* genes.

## Materials and methods

### Plant material and growth conditions

Two spring wheat cultivars (*Triticum aestivum* L.) Kontesa and Ostka were used in the study. Thirty seeds from each cultivar were germinated in wet glass beads in Petri dishes for 2 days at 4 °C, followed by 5 days at room temperature in the dark. Subsequently, 18 seedlings from each cultivar were planted into soil pots and grown in a growth chamber under long-day conditions (16 h of light at 20 °C and 8 h of dark at 18 °C) with a light intensity of 350 µmol m^−2^ s^−1^.

The following samples were collected from each cultivar in three biological replicates: 5-day-old seedling roots, 4-week-old plant leaves (longest, well developed leaves), 5–6 cm long inflorescences, and developing spikes harvested at 0, 4, 7 and 14 days after pollination (0, 4, 7 and 14 DAP). Additionally, flag leaves were collected simultaneously with the inflorescences and spikes (FL inflorescence, FL 0, FL 4, FL 7, and FL 14 DAP).

All collected samples were immediately frozen in liquid nitrogen and stored at − 80 °C.

### Selection of reference genes and validation of RT-qPCR primers

A set of ten candidate reference genes with relatively stable expression in wheat plants was selected based on previous expression studies. These genes, along with references to the studies and sources of primer pair sequences, are listed in Table 1. The specificity of the primers was verified using 2% agarose gel electrophoresis and visualization of RT-qPCR melting curves. All primer pairs amplified a single target product with the correct length and a single peak in the melting curve analysis.

### RNA extraction and cDNA synthesis

Total RNA was extracted from all collected samples using TRIzol Reagent (Invitrogen, Lithuania) following the manufacturer’s protocol. The quality and concentration of the extracted RNA were evaluated using 1.5% agarose gel electrophoresis and a NanoDrop spectrophotometer (NanoDrop ND-1000, Thermo Fisher Scientific, Wilmington, DE, USA). High-quality RNA was reverse-transcribed into cDNA using the RevertAid First Strand cDNA Synthesis Kit (Thermo Scientific, Lithuania), with 4 µg of RNA in a 20 µL reaction volume. The resulting cDNA was diluted 20-fold before use in RT-qPCR assays.

### RT-qPCR conditions

RT-qPCR analysis was performed in three experiments, each using a different set of reference genes and cDNA samples. The first experiment assessed the expression stability of ten candidate reference genes (Table 1, Table S1) in three different organs of the Ostka cultivar: seedling roots, 4-week-old leaves, and 7 DAP spikes. Then six genes (*Actin*, *CPD*, *Cyclophilin*, *Ref 2*, *Ta14126*, *Ta3006*) were selected for the second experiment, which included five different tissues from Ostka: seedling roots, 4-week-old leaves, 7 DAP spikes, 14 DAP spikes, and flag leaves accompanying 14 DAP spikes (FL 14 DAP). In the final experiment, two genes (*Ta3006* and *Ref 2*) were used to analyze expression levels across all samples from both Ostka and Kontesa cultivars. Additionally, the absolute expression levels of two *TaIPT* genes (*TaIPT1* and *TaIPT5*, Table S2) were compared, with normalization performed using one or both selected reference genes. For normalization to two reference genes, the geometric mean of their expression was calculated and used as a normalization factor. The geometric mean, which represents the central tendency of a set of positive real numbers by using the product of their values (as opposed to the arithmetic mean which uses their sum), was calculated for the selected reference genes, following the method of Vandesompele et al.^20^.

All qPCR reactions were carried out on a CFX384 Touch Real-Time PCR Detection System (Bio-Rad Laboratories, Hercules, CA, USA), except for the second experiment, which was performed using the LightCycler 480 Real-Time PCR System (LightCycler 480 II, Roche Diagnostics, Rotkreuz, Switzerland). Each experiment was conducted on a 384-well plate with a reaction volume of 10 µL, consisting of 2 µL of diluted cDNA, 0.2 µM of each primer, and 1× HOT FIREPol EvaGreen qPCR Mix Plus (Solis BioDyne, Estonia). The reactions were carried out in two or three biological replicates and three technical replicates. The temperature profile for all reactions was as follows: initial denaturation/polymerase activation at 95 °C for 12 min; 50 cycles of amplification at 95 °C for 15 s, 60 °C for 20 s, and 72 °C for 20 s; followed by a melting curve analysis from 70 °C to 99 °C with a temperature increase of 0.5 °C/s and continuous fluorescence measurement.

For candidate reference gene evaluation, the average Ct value from three technical replicates was calculated and used in subsequent analyses. For *TaIPT* genes analysis, expression levels were calculated using the standard curve method. The relative expression for *TaIPT1* and *TaIPT5* was determined based on the arithmetic mean expression across all samples.

### Data analysis

PCR efficiency for each primer pair was calculated using LinRegPCR (version 2021.2)^44^ with raw fluorescence data as input.

The stability of candidate reference genes was evaluated using four statistical algorithms: Bestkeeper^24^NormFinder^23^RefFinder^25^ and geNorm^20^. Calculations for Bestkeeper and NormFinder were performed in Microsoft Excel, while RefFinder was conducted using an online-based tool (https://www.ciidirsinaloa.com.mx/RefFinder-master/?type=reference**).** GeNorm analysis was performed using the qbase + software (Biogazelle).

### Statistical analysis

Statistical analyses were performed using Statistica 13.3 software (StatSoft, Krakow, Poland). For Fig. 3, pairwise comparisons between two cultivars were conducted for each tissue separately, without comparing across tissues. Depending on the data distribution (assessed by the Shapiro-Wilk test) and homogeneity of variances (Levene’s test), either the Student’s t-test or Mann-Whitney U test was used. For Fig. 4, absolute and normalized expression values within the same tissue were compared. In this case, one-way ANOVA or the Kruskal-Wallis test was applied, depending on normality and variance homogeneity. When significant differences were detected, appropriate post hoc tests (Bonferroni or Scheffé) were used, both of which account for multiple comparisons and control the Type I error rate.

**Table 1:** List of reference genes, their genbank/ensemblplants accession numbers, and the forward and reverse primers used in this research.

Symbol of reference gene	Gene annotation	GenBank/EnsemblPlants accession number	Forward/reverse primers (5′-3′) used in this research	Preliminary references
*Ref 2/Ta2291*	*ADP-ribosylation factor*	AB050957.1 TraesCS3D02G330500 TraesCS3A02G337300	GCTCTCCAACAACATTGCCAAC GCTTCTGCCTGTCACATACGC	^21^
*Ta3006*	*Wings apart-like protein 2*	XM_044593507 TraesCS1D02G168800	CTGTGGGTCTGTCTAAGAATGCG CAAGTTGTTGTTTGGAAGGCAGC	^26^
*Actin*	*Actin*	KC775782.1 TraesCS1A02G179000 TraesCS1B02G201400 TraesCS1D02G177400	CACACTGGTGTTATGGTAGG AGAAGGTGTGATGCCAAAT	^12,21^
*Ta2776/RLI*	*68 kDa protein HP68*	AY059462 TraesCS4A02G143000	CGATTCAGAGCAGCGTATTGTTG AGTTGGTCGGGTCTCTTCTAAATG	^21,27^
*CPD*	*Cyclic phosphodiesterase-like protein*	AK453202 TraesCS1A02G338500 TraesCS1D02G340700	CGACTTCTTCTACCAGTGCGT GGGTTGATCTCTGAAACCCGA	^31^
*Cyclophilin*	*Peptidyl-prolyl cis-trans isomerase (Cyclophilin)*	AF542973.1 TraesCS7A02G410100	CAGGTCGGGTTGTCATGG TCCCCTTGTAGTGGAGAGGC	^12^
*Ta14126*	*Housekeeping genes encoding the scaffold-associated regions DNA-binding protein*	AK330303 TraesCS7A02G446000 TraesCS7D02G436100	GAGTCTGCCCACCCATTCGTAA GACATGCCATAGGTTTCAGCGAC	^26^
*eF1a*	*Translation elongation factor EF-1alpha (GTPase)*	M90077.2 TraesCS4D02G197200 TraesCS4B02G196800	CAGATTGGCAACGGCTACG CGGACAGCAAAACGACCAAG	^32^ ^12,21^
*GAPDH*	*Glyceraldehyde-3-phosph. dehydrogenase*	KR029492.1 TraesCS6A02G213700 TraesCS6B02G243700 TraesCS6D02G196300	TTCAACATCATTCCAAGCAGC CGTAACCCAAAATGCCCTTG	^32^ ^12,21^
*B-tubulin*	*Beta-tubulin 5 (Tubb5)*	TraesCS1A02G309700 TraesCS1B02G320800 TraesCS1D02G309200	CCATCAGTTGGTTGAGAATGC CAAAGCTGGGAGTGGTCA	^12,21^

## Results

### Experiment 1: ten candidate reference genes tested in three different tissues

**Fig. 1:**
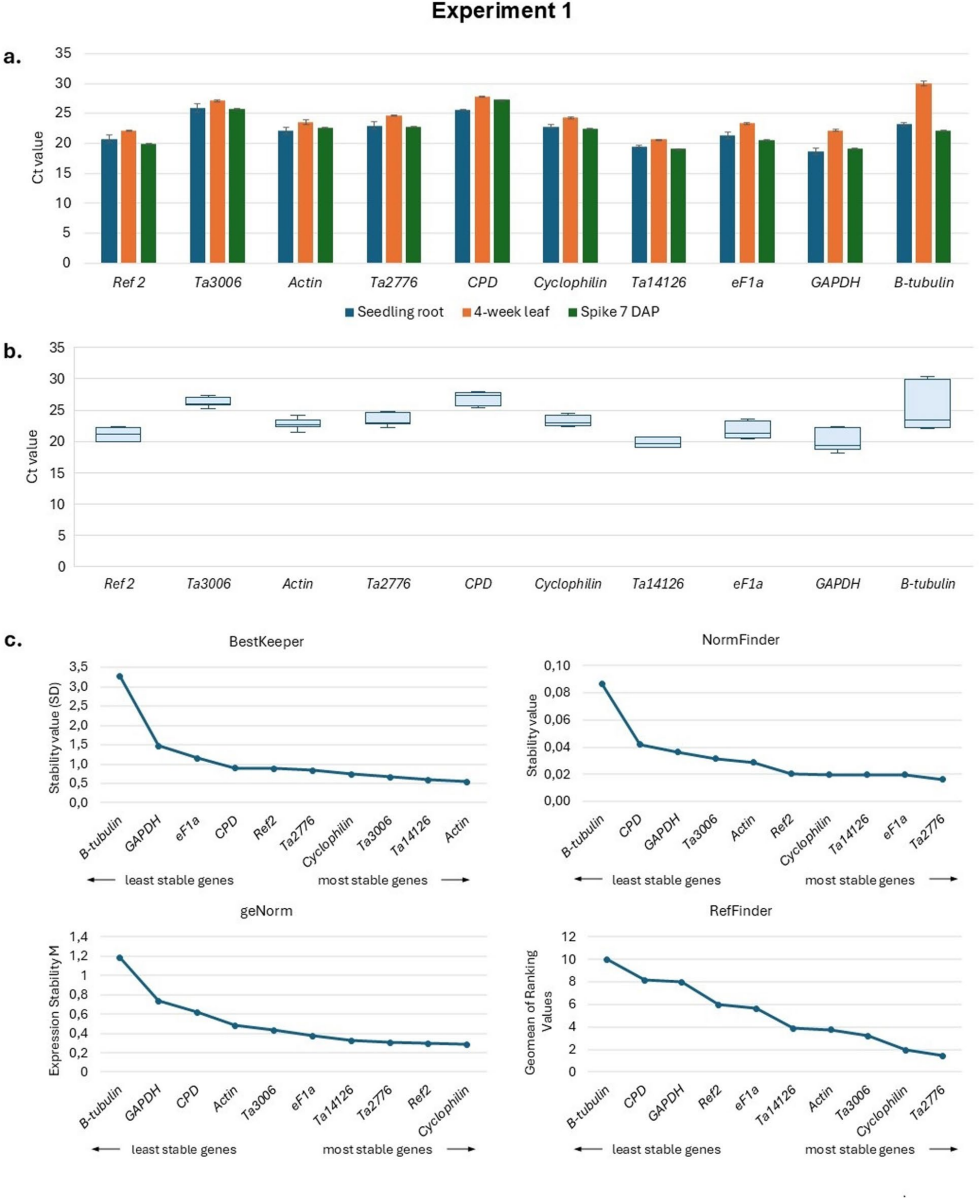
Experiment 1. Expression analysis of ten candidate reference genes in three different tissues of developing wheat plants of Ostka (**a**,**b**) and their expression stability rankings according to four different software programs: BestKeeper^24^NormFinder^23^geNorm^20,45^and RefFinder^25,46^ (**c**). Error bars denote ± SD.

The expression analysis of ten candidate reference genes in three different tissues of developing wheat plants from the Ostka cultivar is presented in Fig. 1a, b and Table S3. Data represent Ct values reflecting expression levels between tissues. Most reference genes were expressed at similar levels in the tested tissues: seedling roots, 4-week leaves, and 7 DAP spikes (7 DAP). However, the *β-tubulin* gene showed the most variable expression in these tissues.

The rankings of expression stability for the ten reference genes, as assessed by four algorithms, are shown in Fig. 1c. BestKeeper evaluates stability by calculating the standard deviation and the coefficient of variation for candidate genes. NormFinder identifies genes with minimal variation both between and within groups. geNorm assesses pairwise variation among genes to determine gene expression stability based on pairwise variation. RefFinder integrates data from the other three tools to provide a comprehensive stability ranking. Based on these rankings, the choice of the most suitable reference genes for normalization in gene expression studies in the three wheat tissues depends in part on the software used. Nevertheless, *β-tubulin*, *CPD* and *GAPDH* were consistently among the least stable genes, while *Ta2776*, *eF1a*, *Cyclophilin*, *Ta3006*, *Ta14126*, and *Ref 2* were among the most stable in all software analyses.

### Experiment 2: six candidate reference genes tested in five different tissues

**Fig. 2:**
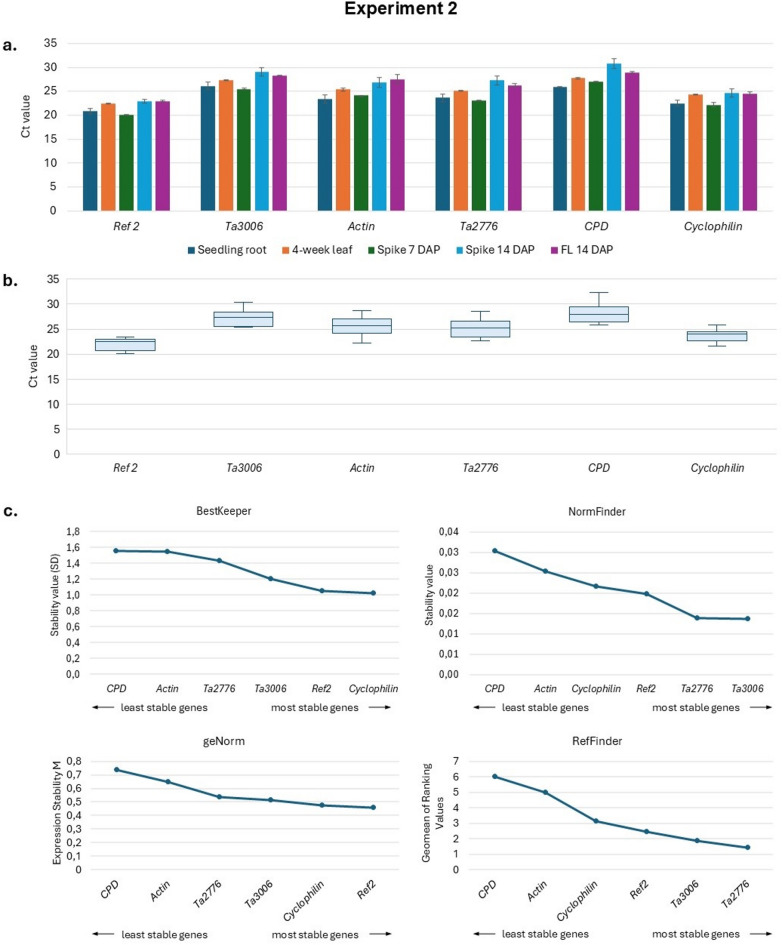
Experiment 2. Expression analysis of six candidate reference genes in five different tissues of developing wheat plants of Ostka (**a**,**b**) and their expression stability rankings according to four different software programs: BestKeeper^24^NormFinder^23^geNorm^20,45^and RefFinder^25,46^ (**c**). Error bars denote ± SD.

In Experiment 2, we analyzed the expression of selected from experiment 1 six candidate reference genes (*Ref 2*, *Ta3006*, *Actin*, *Ta2776*, *CPD*, *Cyclophilin*), showing high expression stability in five different tissues of developing wheat plants from the Ostka cultivar: seedling roots, 4-week leaves, 7 DAP spikes, 14 DAP spikes, flag leaves 14 DAP (FL 14 DAP) (Fig. 2a, b and Table S3). Most reference genes were expressed at relatively consistent levels across these tissues.

The rankings of expression stability for the six reference genes, as determined by the four software programs (Fig. 2c and Table S4), identified *CPD* and *Actin* as the least stable genes. On the contrary, *Ta2776*, *Cyclophilin*, *Ta3006*, and *Ref 2* were among the most stable. The classification of these four genes varied slightly depending on the software used.

### Experiment 3: two candidate reference genes tested in twelve different tissues of two cultivars

**Fig. 3:**
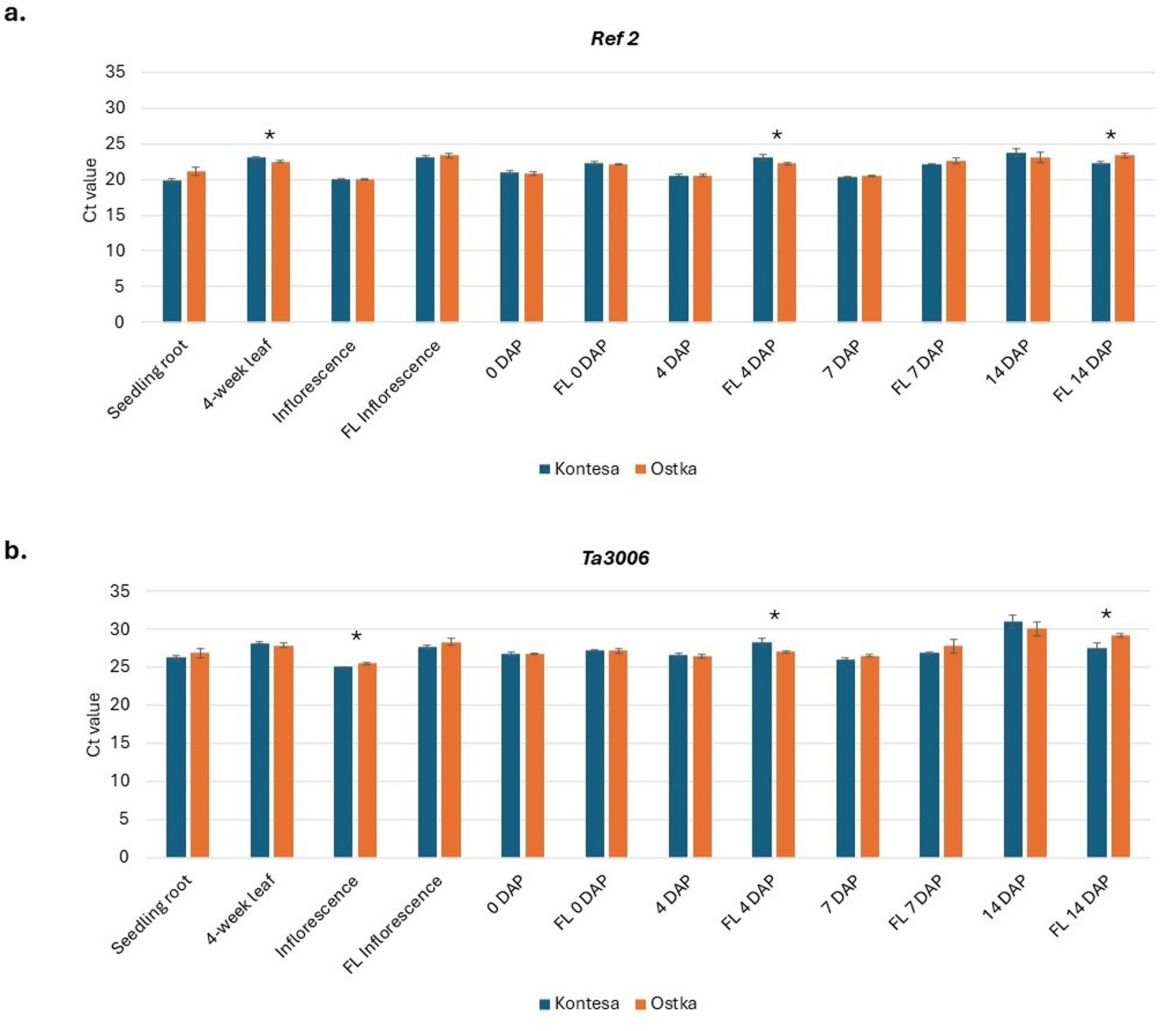
Expression analysis of reference genes *Ref 2* (**a**) and *Ta3006* (**b**) in twelve different tissues/organs of developing wheat plants from two cultivars: Kontesa and Ostka. Error bars denote ± SD. Depending on the distribution normality and homogeneity of variances, either Student’s t-test or the Mann-Whitney U test was used to compare significant differences for candidate gene between cultivars: *significant at *p* ≤ 0.05.

In the next experiment, we evaluated the expression of two selected reference genes, *Ref* 2 and *Ta3006*, in twelve different tissues/organs of developing wheat plants from two cultivars, Kontesa and Ostka (Fig. 3a, b and Table S5). These reference genes showed the most stable expression among the three in the experiment 2, and one of them, *Ref2* has already been used as reference gene in our previous experiments. While very low SD values were observed, no significant differences in expression levels were detected between the two cultivars for most tissues tested. These tissues included seedling roots, 4-week leaves, 0, 4, 7 14 DAP spikes, flag leaves collected during floret initiation (FL inflorescence), and flag leaves 7 DAP during spike development (FL 7 DAP). Significant differences at *p* ≤ 0.05 between cultivars were detected for *Ref 2* in the 4-week leaf, FL 4 DAP, and FL 14 DAP, and for *Ta3006* in the inflorescence, FL 4 DAP, and FL 14 DAP. The Ct values across all the samples ranged from 19 to 24 for *Ref 2* and 25 to 30 for *Ta3006*.

### Comparison of expression values of two *IPT* genes normalized by two reference genes

**Fig. 4:**
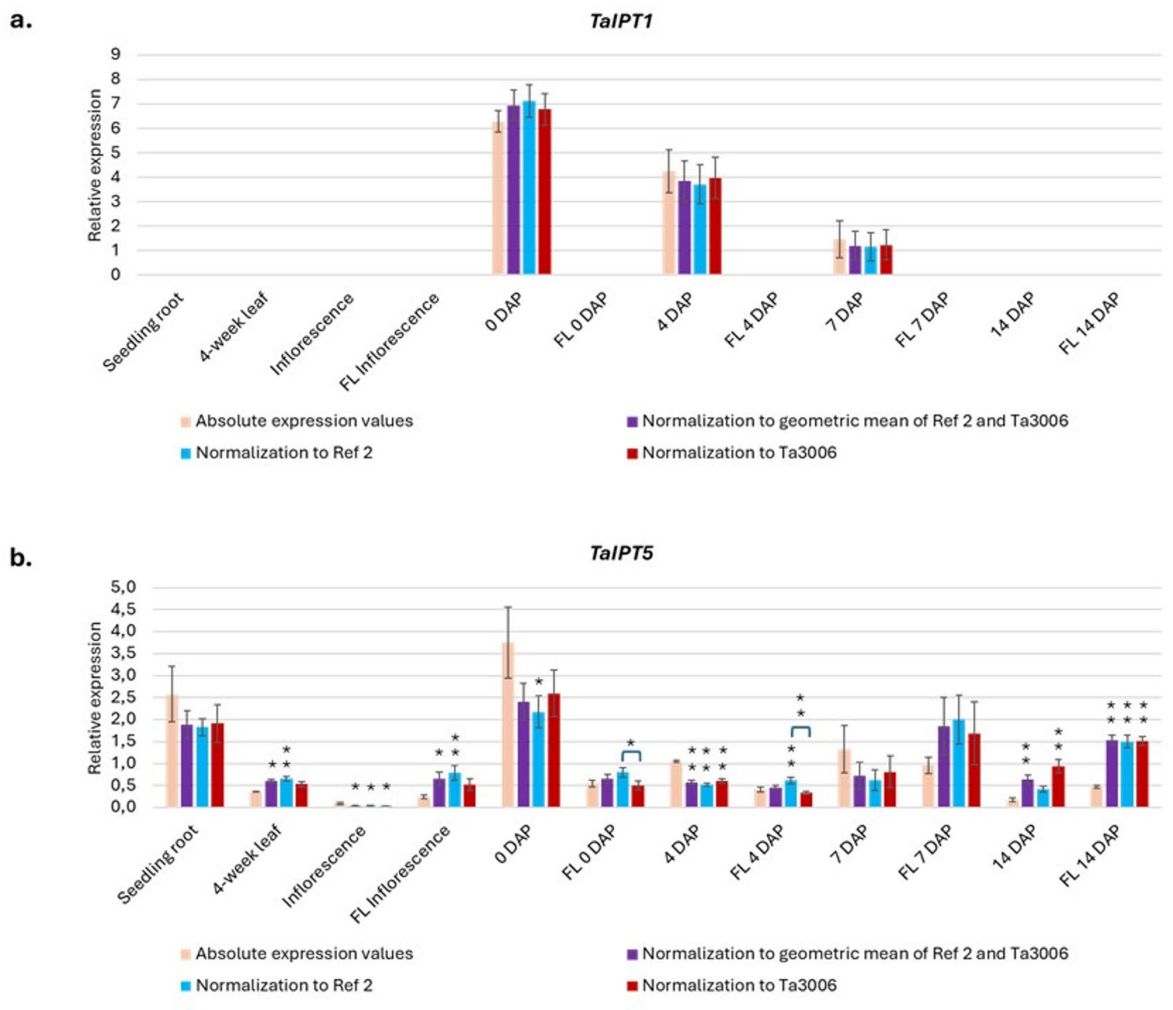
Comparison of the absolute expression values of *TaIPT1* (**a**) and *TaIPT5* (**b**) with their normalized values using *Ref 2*, *Ta3006*, or both reference genes in the Ostka cultivar. Error bars denote ± SE. One-way ANOVA or the Kruskal–Wallis test was applied, depending on the normality of distribution (assessed with the Shapiro-Wilk test) and homogeneity of variances (Levene’s test). Significant differences for *TaIPT1* (not found) and *TaIPT5*: *significant at *p* ≤ 0.05; **significant at *p* ≤ 0.01.

Finally, we compared the absolute expression values of two *IPT* genes (*IPT1* and *IPT5*) with their normalized values using *Ref 2*, *Ta3006*, or both reference genes (Fig. 4a, b and Table S6). *IPT1*, which is specifically expressed in developing spikes (0, 4, and 7 DAP), showed no significant differences between the absolute expression values and normalized values using one or both reference genes (Fig. 4a). In contrast, *IPT5*, which is expressed in all tissues and organs tested, showed significant differences between the absolute expression values and most normalized values (using *Ref 2*, *Ta3006*, or both reference genes) in nearly all tissues (Fig. 4b). However, no significant differences were observed between most expression values normalized with either reference gene alone or combined. Significant differences between normalized values using *Ref 2*, *Ta3006* were detected only for FL 0 and 4 DAP.

## Discussion

Among the most important groups of genes that coordinate plant development are those that regulate phytohormone homeostasis and content. The genes belong to gene families and are expressed specifically in selected organs or spatio-temporally through developing plants. Prior recognition of their patterns of expression in different organs of developing plants is an initial step in characterizing their function.

Quantitative, real-time PCR (qPCR) is among the most precise and reliable techniques for gene expression analysis. However, selecting and validating stable reference genes is crucial for accurate normalization, especially in complex allohexaploid species such as *Triticum aestivum* (common wheat). In our unpublished preliminary research, we identified *ADP-ribosylation factor* (*Ref 2*) as the most suitable reference gene for studying the expression of spatio-temporally regulated gene families. *Ref 2* has been successfully used in previous studies on the expression patterns of *TaCKX* genes^13,41,42^and *TaNAC* genes^43^. However, some researchers have opted to use two or even more reference genes to obtain more comparable results^27^. Therefore, before studying the next family of phytohormone-regulating genes, we conducted research on ten candidate reference genes for their stability of expression in various tissues and developmental stages of wheat, and validated them through rigorous analysis using multiple statistical tools (BestKeeper^24^NormFinder^23^geNorm^20^ and RefFinder^25,46^).

### Identification of stable reference genes

A three-step analysis was conducted to limit the number of experimental trials. In Experiment 1, we evaluated ten candidate reference genes using three very diverse tissues/organs collected at different developmental stages of plant growth. The goal was to pre-select the most suitable and exclude the most unstable reference genes. These ten candidate reference genes were assessed in three tissues of the Ostka cultivar, revealing considerable variation in expression stability. Genes such as *β-tubulin*, *CPD*, and *GAPDH* exhibited high variability and were deemed unsuitable for normalization. Conversely, *Ref 2*, *Ta2776*, *Cyclophilin*, *Ta3006*, and *Ta14126* consistently ranked as the most stable across all software analyses. Similarly, *GAPDH* was identified as the least stable reference gene during endosperm development in wheat, as reported by Mu et al.^28^.

Expanding the analysis in Experiment 2, six selected genes were evaluated in five tissues, further validating *Ref 2*, *Ta3006*, *Ta2776*, and *Cyclophilin* as robust reference genes. The exclusion of *CPD* and *Actin* from this group underscores the importance of tissue-specific testing, as these genes, although widely used in other contexts, proved less stable under the conditions tested here.

Part of these findings is consistent with previous studies highlighting the reliability of *Ref 2/Ta2291*, *Ta2776* and *Cyclophilin* as the most stable reference genes for normalizing gene expression in different tissues and development stages of wheat^21^. In their research, these genes outperformed all traditional housekeeping genes such as *actin* and *α-tubulin*, *β-tubulin*, *Ubiquitin*, and *GAPDH*. Conversely, the authors did not find *Cyclophilin* as stable as in our research. However, the same reference gene was among the two best for studying wheat flag leaf expression in three cultivars grown under different farming conditions^30^. Similarly, *Ref 2* was validated as the most stable gene to study grain filling in wheat^29^. The reliability of this reference gene was demonstrated by monitoring the expression dynamics of three *NAM* genes (*TaNAM-A1*, *TaNAM-B1*, and *TaNAM-B2*) in flag leaves or the 10 samples tested. Their results suggest that this single reference gene, identified by geNormPlus, is optimal for use in this experiment. The *Elongation factor 1 alpha-subunit 2* was the second in ranking, though noticeably less stable than *Ref 2*. *Glyceraldehyde-3-phosphate dehydrogenase*, *translation elongation factor EF-1alpha* (*TEF1*), *CPD*, *actin* and *tubulin* gene families were also used to study wheat meiosis^31^ and in other studies^47^.

Several reference genes that we used to study expression of spatio-temporally regulated genes in different organs of developing wheat plants have also been used in stress response studies^47^. In studies of gene expression under biotic or abiotic conditions, *actin* was the best candidate among housekeeping genes in common wheat seedlings under short-term drought stress^34^. *Glyceraldehyde-3-phosphate dehydrogenase* (*GAPDH*) was ranked among the three most stable internal control genes for studying viral infections in cereals^35^. On the other hand, *elongation factor-1 alpha* for barley and oat samples, and α-*tubulin* for wheat samples, were consistently ranked as the less reliable controls. In durum wheat (*Triticum durum* L.) under drought stress, the most stable reference genes were *GAPDH*, *ubiquitin* and *β-tubulin2*, whereas under salinity stress conditions, the most stable reference genes were *eukaryotic elongation factor 1-α*,* glyceraldehyde-3 phosphate* and *actin*^33^. Scholtz and Visser^36^ documented that there is a need for validation of reference genes for the analysis of the expression of each different plant-pathogen interaction.

Several reference genes tested by us were also validated in other cereal genomes such as hexaploid oat (*Avena sativa* L.)^22^. The most stable for all samples starting from seedling shoots and roots to developing seeds and endosperms was *EIF4A* (*Eukaryotic initiation factor 4 A-3*). The same reference gene *EF1A* (*elongation factor 1‑alpha*) was among the two most stable in in *Avena sativa* during compatible and incompatible interactions with two different pathotypes of *Puccinia coronata* f. sp. *Avenae*^37^. In the same system, *Cyclophilin* was shown to be the worst candidate. *EF1A* (*elongation factor 1‑alpha*) was also one of the three most appropriate reference genes in studies of compatible and incompatible interactions with *Puccinia graminis* in the same cereal species^38^. These differences in the stability of reference genes can be dependent on experimental system and conditions, as well as species specificity and their interaction with pathogens. As documented by Yang et al.^22^ in oat, the feasibility of reference genes for qPCR in polyploid species is influenced by the number of copies of these genes.

### Validation across cultivars and tissues

In the subsequent experiment comparing two wheat cultivars (Kontesa and Ostka), *Ref 2* and *Ta3006* demonstrated consistent expression across twelve tissues, further supporting their suitability as reference genes. This consistency between cultivars enhances their utility in broader genetic and physiological studies of wheat, addressing concerns about genotype-specific variability. The Ct values for *Ref 2* and *Ta3006* in all samples ranged between 20 and 24, and 25 and 30, respectively, reflecting uniform expression levels regardless of tissue type or developmental stage.

### Application to normalization of target genes

The utility of these reference genes was further demonstrated in normalization of *TaIPT1* and *TaIPT5* gene expression. *TaIPT1*, which is specifically expressed in developing spikes, did not show significant differences between absolute and normalized expression values when either *Ref 2* or *Ta3006* was used. This highlights the precision and reliability of these reference genes in quantifying tissue-specific expression patterns. Similarly, for *TaIPT5*, a broadly expressed gene, normalization with *Ref 2*, *Ta3006*, or their combination yielded consistent results, emphasizing their robustness even in complex expression analyses.

### Comparison of single and dual reference genes

Although normalization of *IPT1* and *IPT5* using single or dual reference genes yielded similar trends, the use of two reference genes can enhance precision and reliability, especially in experiments involving multiple tissues or stress conditions. This observation aligns with earlier recommendations for using multiple reference genes in gene expression studies^19–21^. However, our findings are also consistent with a study on grain filling in wheat, where *Ref 2* was identified as an optimal single reference gene^29^.

### Implications, limitations and future directions

The validated reference genes identified in this study, particularly *Ref 2*, *Ta3006*, *Ta2776*, and *Cyclophilin*, provide a robust foundation for RT-qPCR-based expression analyses in wheat. Their consistent performance across tissues, developmental stages, and cultivars underscores their utility in diverse experimental contexts.

This study was conducted under standard growth conditions, using two cultivars, twelve diverse tissues/organs collected at different developmental stages, and ten candidate reference genes. Although two wheat cultivars were analyzed, the findings may not fully represent the genetic diversity of wheat or related Triticeae species. Therefore validation of reference genes in additional wheat varieties and related Triticeae species could further expand their applicability. Further research should also investigate their stability under various abiotic and biotic stress conditions to extend their relevance to studies of stress responses in wheat.

In conclusion, this study identifies and validates four reliable reference genes for RT-qPCR normalization in wheat: *Ref 2*, *Ta3006*, *Ta2776*, and *Cyclophilin*, facilitating accurate expression analysis of developmentally regulated genes. Among them *Ref2* and *Ta3006* had comparable expression levels in different genotypes across the tissues tested, and each is sufficient for normalization of developmentally regulated genes in wheat. Through rigorous analysis using multiple statistical tools (BestKeeper, NormFinder, geNorm, and RefFinder), our findings contribute significantly to optimizing RT-qPCR normalization in wheat. These findings will help researchers characterize gene functions, advancing molecular breeding efforts, and ultimately improve wheat productivity and resilience.

## Electronic supplementary material

Below is the link to the electronic supplementary material.

Supplementary Material 1

## Data Availability

All data generated or analyzed during this study are included in this published article and its supplementary information files.
